# Supplementary material for: Rural Emergency 360°: A mixed-methods analysis of barriers and priorities for equitable emergency care in Québec
Source: PLoS One. 2026 Sep 18;21(9):e0342965. doi: 10.1371/journal.pone.0342965 (PMC13588519; doi:10.1371/journal.pone.0342965)
Supplement: Supplementary file 2 [file pone.0342965.s001.docx]

**Supplementary Appendix 1. Representative Quotations by Theme**

This appendix presents extended participant quotations that support the thematic findings reported in the Results section. Quotations are organized by the four main themes identified during the data analysis (governance, healthcare organization, access to resources, professional practice), along with relevant sub-themes. Participant identifiers are retained for transparency.

**Theme 1: Governance**

**Financial Resources and Funding Models**

- *“Now, what do we do, you know, when we have THAT case that arrives at 3 in the morning? So, then, well, we put everything into perspective: ‘OK, what do we do? Does radiology change anything? Does the laboratory change anything?’ You know, it’s all that, there.”* [EI53_ges]

**Loss of Local Autonomy**

- *“But the moral of the story is also that (…) decisions are no longer made in rural areas. Normally, now, for all the small emergencies in Quebec, there isn’t a decision made at the hospital itself. That’s over. So that’s why everything […] next to it has become very big because it’s part of a machine.”* [FG09_cit]
- *“It should be settled in the same way as elsewhere. ‘Oh! They don’t have that in the laboratories in such a place, so we won’t have it at such a place.’ Pff! Meanwhile, the local population could be better served by another solution that is completely satisfactory, and we… Elsewhere, they’ll justify that it doesn’t apply elsewhere, but here, it could be done.”* [EI05_md]

**Administrative Complexity**

- *“At the emergency level, we still have a listening ear from local managers, and you know, sometimes, it happens that changes are proposed, and efforts are made to keep it from reaching the higher-ups and try to solve it locally so that it moves faster, and that’s still a bit possible. So I think that’s an advantage of being in a small place. […] So the mobilization of a team is still there, but as soon as we start moving up the ranks and end up at the CISSS level, there’s nothing feasible in the short term.”* [EI35_md]

**Standardization versus Local Adaptation**

- *“Then we waste an insane amount of time every three years redoing the protocols, checking them. We are all the same, we don’t have time to take care of the protocols. We pick up the phone, and we call our friends who have worked on them in the big centers and they send them to us. But in practice, it should be done day-to-day in everything. The nutritionists who have made a bunch of good recipes at Laval Hospital and in Montreal for renal insufficiency, cardiac issues, […] everything you can think of, they have all been thought out, these recipes. They should put them on the Internet and share them with the whole network. [Here], when it’s time to feed someone with a severe diabetes problem, they won’t waste time looking for recipes, they will go there.”* [EI02_md]

**Performance Monitoring**

- *“Of course, the demands from the ministry are enormous. I find that it is very focused on statistics, and not on the client, less on the clinical aspect: which we, the social workers, struggle a bit with because we are not stat machines, we help people! […] You know, we report what we do. There are things that don’t fit into the statistics. If I take steps because I have a client who no longer has a fridge, it’s broken […] So then, I take steps with the Knights of Columbus, I call the Help Center. All these steps, even if it took me 3 hours, I can’t report it. […] What they ask us to do for stats is not representative of a social worker’s job. But at the same time, as long as my client is well, the stat doesn’t matter much to me. Except that the government, whether we like it or not, sets targets. So the managers, at some point, have no choice but to say: ‘You have to reach your target.’”* [EI10_psy]
- *“(Health minister) has given orders that patients must not stay more than 20 hours in the emergency room. They must not be... They must be moved when they are admitted. We have two hours to move the patient. If you don’t have a bed upstairs, you put them in the hallway to hide them. It’s awful, it’s terrible! […] And we treat patients by numbers. And we are also treated by numbers. […] It’s because we are outside. Like right now, we are doing a CT scan, but we don’t have a radiologist here. We have to wait until after 9 AM, [the city radiologist] who will read it when he feels like it. And sometimes, we wait 4-5 hours. So, that goes into our emergency stats. It delays us a lot. We end up losing points.”* [FG20_inf]

**Collaborative and Community-Based Governance**

- *“We are even very advanced in terms of control rooms, coherence matrices, and visual stations deployed in the teams. I think that, at the administrative level, this is the link between senior management and field staff. What I mean is that each department, well, obviously, we have deliverables, right, with either the minister’s priorities or the targets of the management agreement, etc., but now, we have deployed this in coherence matrices. […] So you see, there is really a link between the management agreement and the field to say, ‘well, look, team, we have this to do, and in operations, what means are we going to take together?’ Then, well, it goes up in the sense that I am monitoring the indicators in my office. P4, P5 in the emergency room, it’s exactly the same principle.”* [EI27_ges]
- *“Or often, it’s citizen pressure. I’m thinking of hemodialysis; there was a lot of citizen pressure. People who almost let themselves die. There’s a whole media review about it. In short, it took a lot of citizen pressure for it to work. I wouldn’t attribute the success of that solely to the leaders of the CISSS, because often it’s citizen pressure.”* [EI45_cit]

**Theme 2: Organization of Healthcare Services**

**Primary Care and ED Use**

- “As mentioned earlier, we have many patients who no longer have a family doctor. They come in for adjustments to their diabetes medications. That’s not my expertise. It’s not what I work on all day long. […] When we get into the third and fourth lines of hypoglycemic agents […] and so on, often I will seek the opinion of my colleagues or […] a specialist. So, I definitely miss out on that part by not having an office. On the other hand, these are not urgent care needs either. Sometimes we end up doing office work in the emergency department. “[E161_md]
- “In an emergency, it’s crucial to have a doctor present in the ED. However, not every patient who arrives actually needs emergency care—many could be treated at a Family Medicine Group (FMG). But, to retain doctors in the ED, there needs to be a steady volume of patients; otherwise, physicians won’t want to work there. People have become accustomed to relatively convenient service in certain rural Eds where waiting times are generally short and care is immediate. So, while the ideal model would be placing the right patient in the right setting, it’s often hard to implement due to the culture and the need to maintain sufficient activity in the ED to keep doctors engaged.” [GD29_ges]
- *“My principle is having the right person in the right place at the right time. If you’re someone who requires emergency care, you’re in the right place, being cared for by the right people. If you’re someone who doesn’t require emergency care, you’re not in the right place. I actually did a short interview on the radio yesterday for (our region) because we were talking about our small emergencies (CLSC), […] where I have very low patient traffic. For instance, at night, I’ll give you an example, it’s about one person every 4-5 days. That’s not a lot. Now, people are concerned, will they shut down the emergency services 24/7? I don’t see it that way. […] you can maintain your services, but at the same time, what needs to be looked at in terms of resources is, are we using our resources optimally? For example, instead of nurses having nothing to do, can I give them tasks so that they can be utilized effectively and also available for service to the population? […] Well, it could be, for example, setting up processes. […] telemedicine. […] maybe people can also respond to Info-Santé. We’re working on a lot of things. We’re quite creative in keeping our resources and at the same time, ensuring that our emergency performance remains good, and services continue for the population.” [EI25_ges]*

**Interfacility Transfers**

- *“They [the larger centers] don’t know what our reality is, and often, it creates a communication problem between people, between centers. Over time, it’s like, ‘Not another transfer from…’ […] It’s like with polytraumas […] when they arrive there, and we realize there’s not much going on, we say, ‘But why was he transferred?’ But he was transferred because we can’t take any chances.”* [EI16_spe]

**Prehospital Emergency Services**

- *“Because here, we don’t have an airport […] Helicopter service could be a very interesting service; in fact, they have it in several provinces. In Quebec, it’s a closed door, but it could be helpful […] for trauma services and urgent transfers.”* [EI02_md]

**Theme 3: Access to Resources**

**Equipment and Infrastructure**

- *“I talked about it precisely; I was telling [the MSSS], ‘well, require institutions, my RUISSS...’… he asked me to develop two telehealth services this year… if he required external institutions, experts, tertiary institutions, well, it would definitely help me… So, I'm the one who has to knock on all sorts of doors, my medical director has to knock on all sorts of doors.”* [EI28_ges]

**Diagnostic Resources**

- *“If we want to maintain laboratories in places like this, we shouldn't centralize everything… bring back elective tests… to keep technicians focused on laboratory techniques rather than packaging boxes… specimen transport is happening anyway.”* [EI71_cit]

**Medical and Paramedical Specialties**

- *“There are long delays [at the hospital in city X] for elective surgeries… we do office work, private practice in [the city, and they] transfer the patients to us, and we operate on them at [the rural hospital]… It keeps us busy, gives experience to the doctors, anesthetists, nurses… our operating room team.”* [EI62_spe]
- *“How is it possible elsewhere and not possible in Quebec?… If they are capable of offering services to cover the entire specialty within the province, why don't they do it? And if they are unable to do so, why don't they accept solutions that exist in other provinces and that work?”* [EI71_cit]
- *“We have the example of anesthesia where it happened in recent weeks that we didn't have an anesthetist for a while [...] When that happens, it requires mobilizing, I would say, a lot of resources, including pre-hospital services and nursing teams to ensure that we can offer a certain level of safety to the population. [...] I don't know if it's temporary, but it's essential to maintain specialized teams, basic specialties, whether it's surgery, anesthesia, in some cases obstetrics, gynecology, so that's a challenge, indeed. Whether it's maybe a little easier to get temporary assistance or whether we organize differently within the CISSS, but when there are shortages, it's quite spectacular the resources or mobilization that it takes for all sectors of the healthcare network*.” [GD39_diag].
- *“Within the same CISSS, we think... well, they should be able, within their staff, to help each other out. But they are indeed in a situation where several of them are short-staffed. So, the second step we were talking about, well, is it possible then for a center that is not as far away... to help or support them in this work? So, there is work to be done within a CISSS itself, it's not all settled, and then there is also with a more well-equipped peripheral center, even if it's not part of the CISSS.”* [GD29_ges].

**Human Resources**

- *“I'm reaching my limit, but I want the young ones... after five years, 40% of the young ones leave the profession… especially with the mandatory overtime, it's really crazy… they even choose to reduce their salary just to have a better quality of life.”* [EI48_inf]
- *“So, as much as the dynamic with our regular team is enjoyable… at night, with a nurse searching for her things and not knowing where to go, that's not cool.”* [EI03_md]
- *“I will take care of my emergency rooms. They're operating… we keep them all 24/7… trying to keep it open 24/7… it’s much more acceptable.”* [EI53_ges]
- “It's impossible to build a sense of belonging and comfort in a department when you're constantly rotating, with shifts changing three times in a two-week period.”[EI48_INF]

**Theme 4: Professional Practice**

**Skill Maintenance**

- *“We had meetings... about the difficulties or potential shortages in anesthesiology. And the department head [of a university hospital] clearly stated during the meeting that practicing in rural areas for an anesthesiologist was a professional desert. This message resonated within the academic community. So, you know, we brought it back to order by saying that an anesthesiologist, for example, practicing in Gaspé (658 km from Quebec City (major trauma and academic center), well, had a probably much more varied practice, was required to maintain many more skills than the anesthesiologist who is confined to the same type of surgeries for 20 years at Laval Hospital.”* [EI26_ges]

**Professional Isolation**

- *(none reported here beyond main text)*

**Social Proximity and Boundaries**

- “You're following a patient for an alcohol problem, who is in addiction treatment, and theoretically, they're abstinent. Then, you come face to face with them holding a beer. It's like... ‘Hello!’ And then, they look at you, then at their beer, back at you, and back at their beer. […] That's part of the risk, you know.” [EI03_md]
- *“We provide much more personalized care, you know, that's the image. I think we provide care as Florence Nightingale really envisioned it back then, you know. Whereas in a large center, when you have fifteen patients, well, you don't have time to... you know, I'm not saying they provide bad care, but we are a bit more rough on the finishing touches next to it.”* [EI30_ges]

**Continuing Education, Simulation, and Debriefing**

- *“I'm also involved in evaluating the files of trauma patients we receive at the hospital. So, I'm sort of an evaluator for that, and afterward, we sit down with the surgeons, anesthesiologists, and all the doctors from our hospital, and we provide feedback on the trauma cases we have here. Whether it's positive or negative, we do a bit of event reconstruction, and we discuss it with the aim of improving.”* [EI17_inf]

**Training**

- *“But there's an emergency congress in Escoumins that is in a rural area, and often, that one is more aligned with our practice. It's interesting because the solutions they discuss are in rural areas. In Escoumins, there are even fewer resources than we have, and it puts us in a real context of solutions to apply. The conferences really resonate with us more. At least there are people who experience the same reality as us, and that helps.”* [EI36_md]
- **Teamwork**

*“We go upstairs, we cross paths with them [the doctors], we can talk to them in the emergency room if we have a situation that we're not sure whether to bring in or not. The doctors are open. They know us, everyone knows each other. And for clients who are also hospitalized, we have a meeting once a week with the hospital doctor, the nurse, occupational therapist, physiotherapist, nutritionist, and social worker. So, all hospital discharges are organized as a team. Everyone gives their perspective: where we are at, what services we provide. The multidisciplinary work here is really impressive.”* [EI10_psy]
